# Targeting PYK2, entrectinib allays anterior subcapsular cataracts in mice by regulating TGFβ2 signaling pathway

**DOI:** 10.1186/s10020-024-00921-9

**Published:** 2024-09-27

**Authors:** Xuefei Ding, Xiaohe Li, Rui Fang, Peilin Yue, Yuxuan Jia, Enjie Li, Yayue Hu, Honggang Zhou, Xudong Song

**Affiliations:** 1 Beijing Tongren Hospital, Beijing, 100730 China; 2 Capital Medical University, Beijing, 100730 China; 3 Beijing Tongren Eye Center, Beijing, 100730 China; 4 Beijing Ophthalmology & Visual Sciences Key Lab, Beijing, 100730 China; 5 The State Key Laboratory of Medicinal Chemical Biology, College of Pharmacy and Key Laboratory of Molecular Drug Research, Nan Kai University, Tianjin, China

**Keywords:** Fibrotic cataract, EMT, Entrectinib, TGFβ2, PYK2

## Abstract

**Background:**

Fibrosis cataract occurs in patients receiving cataract extraction. Still, no medication that can cure the disease exists in clinical. This study aims to investigate the effects and mechanisms of Entrectinib on fibrotic cataract in vitro and in vivo.

**Methods:**

The human lens cells line SRA 01/04 and C57BL/6J mice were applied in the study. Entrectinib was used in animals and cells. Cataract severity was assessed by slit lamp and Hematoxylin and Eosin staining. Expression of alpha-smooth muscle actin, fibronectin, and collagen I were examined by real-time quantitative PCR, western blotting, and immunofluorescence. Cell proliferation was evaluated by Cell Counting Kit-8. Cell migration was measured by wound healing and transwell assays. Molecular docking, Drug Affinity Responsive Target Stability, and Cellular Thermal Shift Assay were applied to seek and certify the target of Entrectinib treating fibrosis cataract.

**Results:**

Entrectinib can ameliorate fibrotic cataract in vitro and in vivo. At the RNA and the protein levels, the expression of alpha-smooth muscle actin, collagen I, and fibronectin can be downgraded by Entrectinib, while E-cadherin can be upregulated. The migration and proliferation of cells were inhibited by Entrectinib. Mechanistically, Entrectinib obstructs TGFβ2/Smad and TGFβ2/non-Smad signaling pathways to hinder the fibrosis cataract by targeting PYK2 protein.

**Conclusions:**

Targeting with PYK2, Entrectinib can block TGF-β2/Smad and TGF-β2/non-Smad signaling pathways, lessen the activation of EMT, and alleviate fibrosis cataract. Entrectinib may be a potential treatment for fibrosis cataract in clinic.

## Introduction

Mostly, fibrotic cataracts typically appear as anterior subcapsular cataracts (ASC) and posterior capsular opacification (PCO) (Xiong et al. 2022). Lens epithelial cells (LEC) are monolayer cuboidal epithelial cells. Normally, LECs adhere to the inner side of the anterior capsule and maintain lens transparency (Liu et al. 2023; Berthoud et al. 2014). Nevertheless, under some circumstances, such as injury and postoperative, the LECs can be stimulated and epithelial-mesenchymal transformation (EMT) occurs, resulting in fibrotic cataract(Taiyab and West-Mays 2022; Imaizumi et al. 2019). TGFβ2 is the key cytokine that promotes EMT in LECs(Liu et al. 2021; Gotoh et al. 2007). Despite recent research focusing on blocking TGFβ2 signaling pathways to treat lens fibrosis (Li et al. 2022; Wang et al. 2021; Sugiyama et al. 2021), there are still no drugs in clinical application to alleviate the disease.

In clinic, PCO, impairing patients’ vision and decreasing their quality of life seriously, is the most common complication for patients who have undergone phacoemulsification surgery (Wormstone et al. 2021). As society ages and cataract surgery becomes more popular, the number of cataract patients and operative rates continue to climb (Kanasi et al. 2016; Wang et al. 2016). As a result, the incidence of PCO is rising. However, Nd: YAG laser is still the only way to treat PCO, which can lead to complications such as macular edema, intraocular lens damage, and so on (Maedel et al. 2021; Zhang et al. 2022a, b). Therefore, it is necessary to explore an effective drug treatment for lens fibrosis.

Entrectinib is a small-molecule oral medication that was approved by the FDA in 2019 for the treatment of some solid tumors and non-small cell lung cancer in clinic (Al-Salama et al. 2019). Previous clinical studies have demonstrated that Entrectinib is well tolerated and safe while effectively inhibiting tropomyosin receptor kinases A/B/C (Trk A/B/C) (Doebele et al. 2020; Drilon et al. 2020; Demetri et al. 2022; Dziadziuszko et al. 2021; Frampton and Entrectinib 2021). Activation of Trk A/B/C can initiate an EMT program and increase tumor cell proliferation and migratory capacity (Fan et al. 2023; Moriwaki et al. 2022; Jin 2020). Furthermore, it is well-known that organ fibrosis is also closely related to EMT (Su et al. 2020). Some scholars have investigated the therapeutic effects of Entrectinib on pulmonary fibrosis and demonstrated that it could attenuate pulmonary fibrosis in mice (Miao et al. 2022). However, the efficacy of Entrectinib in lens fibrosis has not been reported. Here, we evaluated the role of Entrectinib in lens fibrosis in vitro and vivo.

## Materials and methods

### Cell culture and treatment

Immortalized human lens epithelial cells(ihLECs) SRA 01/04, a commonly used representative cell line in the study of lens diseases, was obtained from American Type Culture Collection (ATCC) and validated by short tandem repeat (STR) analysis. Entrectinib and Protease Inhibitor Cocktail were obtained from Target Mol Co. ltd. (China). Recombinant human TGFβ2 was purchased from MedChemExpress Co., ltd. (America). The cells were cultured in Dulbecco’s Modified Eagle’s medium (DMEM, Solarbio, China) supplemented with 10% (v/v) Fetal Bovine Serum (FBS, Gibco, USA) at 37 °C in a 5% CO_2_ incubator.

### Wound healing assay

Before being scratched with pipette tips, ihLECs were seeded in 24-well plates and incubated for 24 h at 37℃. Then, the debris was washed away by phosphate buffer saline (PBS). And the cells were cultured in DMEM containing 0.1% FBS with TGFβ2 and Entrectinib (0.25, 0.5, 1, 2µM). The wound images were recorded at 0, 6, 12, and 24 h by an inverted optical microscope. The migration rates of scratches at every time point were calculated.

### Transwell assay

24-well filter plates (Corning, USA) with 8 μm pores were utilized to evaluate the vertical migration ability of the cells after different treatments. The cells treated by agents were resuspended with DMEM containing 0.1% FBS and counted by an automated cell counter (Ruiyun Biotechnology Co., Ltd, Shanghai, China). 4 × 10^4^ cells were added in each upper chamber and 600µL 10% serum-containing DMEM was added into the lower chamber. After 24 h, the cells were fixed with 4% paraformaldehyde (PFA) for 30 min. Then the fixed cells were stained with crystal violet for at least 15 min. The cells still in the upper chambers were removed. An inverted microscope was used to photograph the image of the filter. Each filter should be reserved 3 images.

### Cells proliferation and cytotoxicity

Cell proliferation and cytotoxicity were estimated by Cell Counting Kit-8 assay (CCK-8; Solarbio; Shanghai; China). SRA 01/04 cells were cultured in DMEM with 10% FBS in 98-well plates (1 × 10^4^ cells/well) for 24 h. For the proliferation test, the culture media was displaced by DMEM containing 0.1% FBS with TGFβ2 and different doses of Entrectinib. For cell cytotoxicity, the culture media was displaced by DMEM containing 10% FBS and different doses of Entrectinib. After 24 h incubation, CCK-8 assay reagent was added to 96-well plates. A microplate reader measured the optical density (OD) value at 450 nm after 1.5 h incubation. The ratio of cells that survived reflects cell viability (%).

### Immunoblot assay

GAPDH (1:1000, #AF7021), α-SMA(1:1000, #AF1032), collagen I (1:1000, #AF7001), fibronectin (1:1000, #AF5335), E-cadherin (1:1000, #AF0131), P-P38 (1:1000, #AF4001)/ P38 (1:1000, #AF6456), P-ERK (1:1000, #AF1015)/ ERK (1:1000, #AF0155), P-JNK (1:1000, #AF3318)/ JNK (1:1000, #AF6318), P-Smad2 (1:1000, #AF8314)/ Smad2 (1:1000, #AF6449), P-Smad3 (1:1000, #AF8315)/ Smad3(1:1000, #AF6362), and P-pan AKT1/2/3 (1:1000, #AF0016)/pan-AKT1/2/3 (1:1000, #AF6216) antibodies were purchased from Affinity (USA). P-PYK2 (1:1000, #AP2014) antibody was purchased from ABclonal Technology Co., Ltd. (China). PYK2 (1:1000, #14970) antibody was purchased from UpingBio Technology Co., Ltd. (China). In brief, cells were seeded into 6-well plates at a density of 3 × 10^6^ cells/well. Following agent treatments, ice-cold radioimmunoprecipitation (RIPA, Sangon Biotech, China) buffer and Protease Inhibitor Cocktail were applied to lyse the cells or sheared lens tissues. Quantified by BCA Protein Quantification Kit (Yeasen, China), 20 µg protein of each sample was electrophoresed with 8% or 10% SDS-polyacrylamide gel electrophoresis (SDS-PAGE) and transferred to a PVDF membrane. The membrane was saturated in 5% fat-free milk and then incubated in primary antibodies at 4℃ overnight. On the second day, the membrane was washed with TBS containing 0.1% Tween20 (TBST) and then incubated with secondary antibodies for 2 h at room temperature. After 3 times of TBST washing, the bands of the membrane were visualized by Chemiluminescence reagent (Affinity, China). The images were analyzed by Image J.

### Immunofluorescence

For tissue, sodium citrate solution was used for antigen repair of paraffin sections. For cells, the cell slides were fixed in Immunol Staining Fix Solution (Beyotime, China) for 10 min. Then, the tissue and cell slides were permeabilized with 0.2% Triton X-100 (Solarbio, China) for 10 min and incubated in 5% BSA for 1 h at room temperature. The slides were incubated with α-SMA antibody (#AF1032, Affinity, USA) diluted 1:200 in 5% BSA overnight at 4℃. The slides were then incubated with a secondary antibody conjugated by Alexa Fluor 555 for 1 h at room temperature. After cell nuclei were stained with DAPI (Solarbio, China), the cells and tissue slides were observed and snapped by ZEISS LSM 800 confocal microscope and Leica INFINITY TRIF microscope respectively.

### Molecular docking

Forecast through the Super-PRED database(https://prediction.charite.de/index.php), PYK2 was selected as a potential target. After water deletion and hydrogenation, PYK2 (PDB ID:4eku) and Entrectinib were docked by AutoDock Vina 1.1.2 software and Pymol 2.6 software was used for the visualization of docking results.

### Drug affinity responsive target stability (DARTS), cellular thermal shift assay (CETSA) and microscale thermophoresis (MST)

Cell lysis was centrifuged to obtain a soluble protein liquid, and then the solution was divided equally into two samples and incubated, respectively, with Entrectinib (10mM) or DMSO for 2 h at room temperature. For DARTS, different concentrations of pronase (Roche, America) were added into the system for another 30-minute incubation. For CETSA, after heating the samples for 6 min in a metal bath with different temperatures, centrifuge again and separate soluble protein liquid. Finally, western blot was applied to verify the different content of PYK2 in different samples. MST is a technique that tests the binding affinity between two molecules by using the thermophoresis phenomenon (Deeb et al. 2022). In brief, after dyeing with N-hydroxysuccinimide, the recombinant human PYK2 protein (Solarbio, China) was diluted and mixed with Entrectinib of different concentrations. MST capillaries were applied to load the samples and then the capillaries were put into the MST instrument. The data was analyzed with MO. Affinity Analysis software.

### RNA isolation and quantitative real-time polymerase chain reaction (qRT-PCR)

The total RNA of cells or lens tissues was extracted with TRIzol reagent (Solarbio, Beijing, China). According to the manufacturer’s instructions, the RNA was reverse transcribed into cDNA using 5×Hifair^®^ One Step RT SuperMix and gDNA remover (Yeasen, China). The SYBR Green Master Mix (Yeasen, China) was applied to qRT-PCR. The relative expression level was analyzed with 2 − ΔΔ CT method. Primers used in this study are listed in Table 1.

**Table 1:** Primers were applied in this study

Gene	Forward Primer Sequence (5′-3′)	Reverse Primer Sequence (5′-3′)
human GAPDH	GGAGTCCACTGGCGTCTTCA	GTCATGAGTCCTTCCACGATACC
human ACTA2	CCCCAATCGGAAGCCTAACT	GCTGGAAGGTAAACTCTGGATTAGA
human COL1A1	CCAGAAGAACTGGTACATCAGCA	CGCCATACTCGAACTGGAATC
human FN1	GAGCTGCACATGTCTTGGGAAC	GGAGCAAATGGCACCGAGATA
human CDH1	GGGGTCTGTCATGGAAGGTG	CAAAATCCAAGCCCGTGGTG
mouse GAPDH	AGGTCGGTGTGAACGGATTTG	TGTAGACCATGTAGTTGAGGTCA
mouse ACTA2	GCTGGTGATGATGCTCCCA	GCCCATTCCAACCATTACTCC
mouse COL1A1	AACCTAACCATCTGGCATCTCC	GTTTCCAGTCTGCTGTGACCCT
mouse FN1	GTGTAGCACAACTTCCAATTACGAA	GGAATTTCCGCCTCGAGTCT
mouse CDH1	ATTTTTCCCTCGACACCCGAT	TCCCAGGCGTAGACCAAGA

### siRNA transfection

In brief, cells were seeded in the 6-well plates at an appropriate density. Until the cell fusion reached 80%, the transfection regent (TransIT-X2, Mirus, America) and siRNA (Tsingke Biotech, China) complex was applied to transfected the cells with FBS-free DMEM for 48 h. Then, the transfected cells were treated with TGFβ2 and Entrectinib for another 24 h. The transfection efficiency was evaluated by western blotting. The siPYK2 and siNC sequences are displayed in Table 2.

**Table 2:** Sequences of siRNAs applied in this study

siRNA	Sequence (5′–3′)	
	sense	antisense
siPYK2	GGAUCAUCAUGGAAUUGUA	UACAAUUCCAUGAUGAUCC

### Animal experiment

C57BL/6J mice (7 weeks old, 20–25 g) were purchased from Weitong Lihua. Experimental Animal Technology Co., Ltd. (Beijing, China). All animal procedures in the study followed the Association for Research in Vision and Ophthalmology Statement for the Use of Animals in Ophthalmic and Vision Research. The study was approved by the Institutional Animal Care and Use Committee (IACUC) of Nankai University (Permit No. 2023-SYDWLL-000611).

**Fig. 1:**
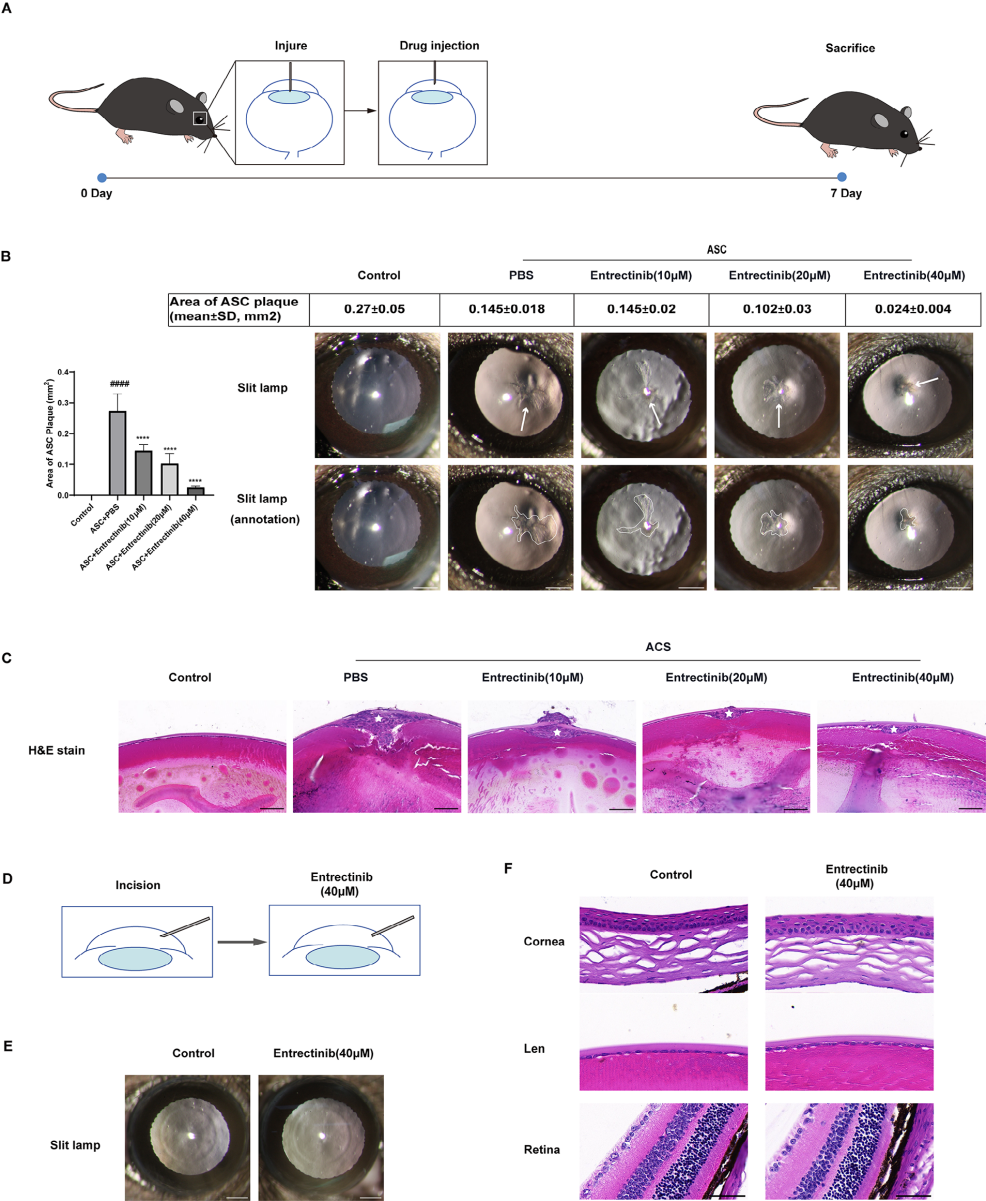
Entrectinib alleviates injury-induced lens fibrosis in mice without appreciably harming the surrounding tissues. (**A**) The ASC mouse model establishment process. (**B**) Typical anterior chamber images of mice treated with or without Entrectinib were obtained via slit lamp (Scale bar = 500 μm). White arrows highlight the typical lesion. (**C**) H&E staining pictures of local fibrosis focus (Scale bar = 100 μm). White pentagrams presented the typical lesion. (**D**) Diagrammatic illustration of the experimental procedure conducted to verify toxicity in vivo. (**E**) Representative anterior segment photography of toxicity validation mice recorded by slit lamp (Scale bar = 500 μm). (**F**) Comparison of intraocular morphology between the mice with Entrectinib treatment and without treatment (Scale bar = 100 μm). The area of fibrosis in the anterior subcapsular was quantified. Data was expressed as means ± SD, *n* = 3. ^####^*P* < 0.0001 versus control group. ^****^P < 0.0001 versus ASC group

**Fig. 2:**
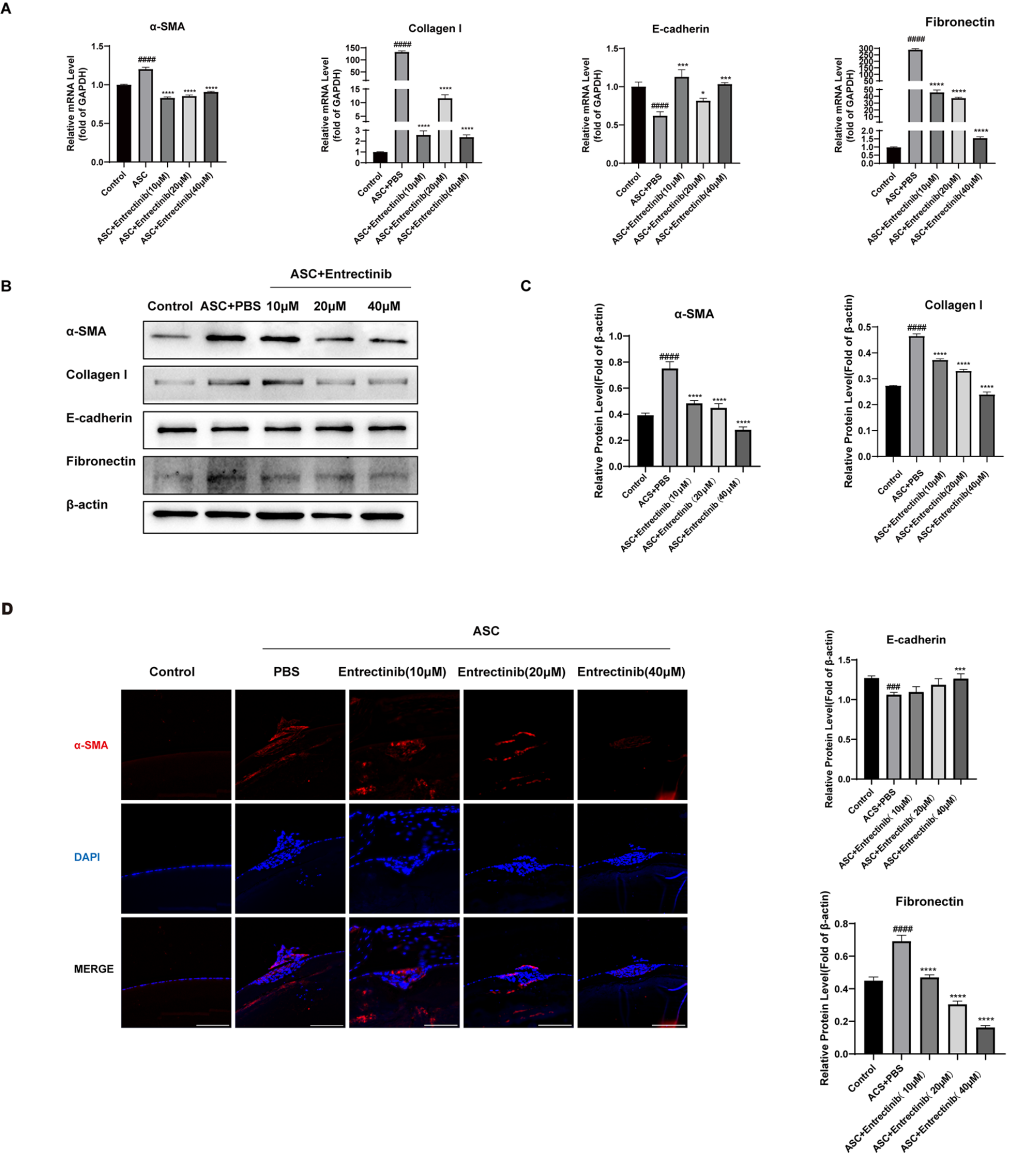
Entrectinib dampens the progression of EMT in ASC mouse models. (**A**) Relative mRNA expression of α-SMA, collagen I, fibronectin, and E-cadherin in mice was analyzed by quantitative real-time PCR. GAPDH served as a reference gene. (**B**-**C**) Western blot was applied to analyze the protein expression of α-SMA, collagen I, fibronectin, and E-cadherin in mice. β-actin served as a reference protein. (**D**) The expression of α-SMA in anterior capsules of mice was evaluated by IF (Scale bar = 200 μm). Data was expressed as means ± SD, *n* = 3. ^**###**^*P* < 0.001 and ^**####**^*P* < 0.0001 versus control group. ^*^P < 0.05, ^***^P < 0.001 and ^****^P < 0.001 versus ASC group

Injury-induced ASC was a common model for investigating lens fibrosis (Fig. 1A). In brief, before surgery, mice were anesthetized with 5% pentobarbital sodium (50 mg/kg) by intraperitoneal injection, then the eye surface was treated with compound tropicamide and proparacaine hydrochloride eye drops. Next, a 26-gauge needle punctured the anterior subcapsular from the central corneal until the 1/4 length (approximately 300 μm) of needlepoint had been into the anterior subcapsular. 1µL PBS or Entrectinib (10, 20, 40µM) were immediately injected into anterior chambers with a microsyringe (Hamilton, 33-gauge) through the central corneal incision. The mice were divided into the control group, the model group, the low-dose administration group, the medium-dose administration group, and the high-dose administration group (10µM, 20µM, 40µM Entrectinib). To assess the toxicity of Entrectinib, a 26-gauge needle was applied to make a cornea incision without damaging other intraocular tissues. Then, 1µL of 40µM Entrectinib was injected into anterior chambers with a microsyringe (Fig. 2D). Ofloxacin Eye Ointment (Shenyang Xingqi Eye Medicine Co., Ltd, China) was applied immediately to avoid postoperative infection. The anterior segment of the eyes was photographed with a slit lamp 7 days after injury. The mice were then dissected and sampled.

### Histological examination

The separated eyeballs were fixed with FAS eyeball fixative solution (Gene-Protein Link, China) for 24 h, then embedded in paraffin after being dehydrated. Until the paraffin is solidified, the sample is sliced at a thickness of 5 μm and stained with hematoxylin-eosin (H&E). The stained tissues were recorded and analyzed by a Pathological section scanner (3DHISTECH, Hungary).

### Statistical analysis

Statistical analysis was performed with SPSS 26.0 (SPSS. Inc., USA) or Graph Prism 8.0 (GraphPad Software, Inc., USA). The quantitative data were presented as mean ± SD. One-way analysis of variance (ANOVA) was used to evaluate the differences between groups. *P* < 0.05 was considered statistically significant.

## Results

### Entrectinib alleviates the fibrotic cataract in injury-induced mouse models

Entrectinib (10µM, 20µM, and 40µM) was injected into the anterior chamber of injury-induced ASC animal models to validate its effectiveness in treating fibrotic cataract. Mice were given 7 days to heal. Then we examined the status of fibrosis lesions in the mouse anterior capsule using a slit lamp (Fig. 1B). Compared with the model group, the fibrosis area of the anterior capsule of the mice treated with Entrectinib is smaller. In addition, the mice with a higher dose of Entrectinib exhibited a milder severity of fibrosis in the anterior subcapsular. The area of lens fibrosis in mice was quantified by Image J. Obviously, the fibrosis area of the mice treated with Entrectinib was substantially smaller in size compared to the model group. As demonstrated by H&E staining results, in the model and treatment groups, the obvious fibroblasts and extracellular matrix (ECM) have been deposited on the wound made 7 days before (Fig. 1C). To estimate the drug toxicity of Entrectinib, 1µL of 40µM Entrectinib was injected into the anterior chamber and the anterior segments were observed with slit lamp and H&E stain 7 days after injection. As Fig. 1E-F demonstrates, Entrectinib at this concentration has no toxic effect on the mice’s cornea, lens, and retina.

### Entrectinib prevents the EMT in ASC mouse models

To assess the anti-EMT effects of Entrectinib in vivo, the lenses were collected to detect the expression of epithelial and fibrosis-related phenotypic markers. ASC mice treated with Entrectinib showed a significant decrease in the expression of α-SMA, collagen I, and fibronectin at both the mRNA (Fig. 2A) and protein levels (Fig. 2B-C) compared to those without Entrectinib treatment. Meanwhile, E-cadherin expression at mRNA and protein levels increased in the ASC mice treated with Entrectinib, which means Entrectinib can recover the reduction of epithelium marker actuated by injury (Fig. 2A-C). The results of immunofluorescence were similar. Compared to the model group, the protein expression of α-SMA at fibrotic tissues in the administration group decreased significantly in a dose-dependent manner (Fig. 2D).

### Entrectinib suppresses TGFβ2- stimulated ihLEC migration and proliferation

**Fig. 3:**
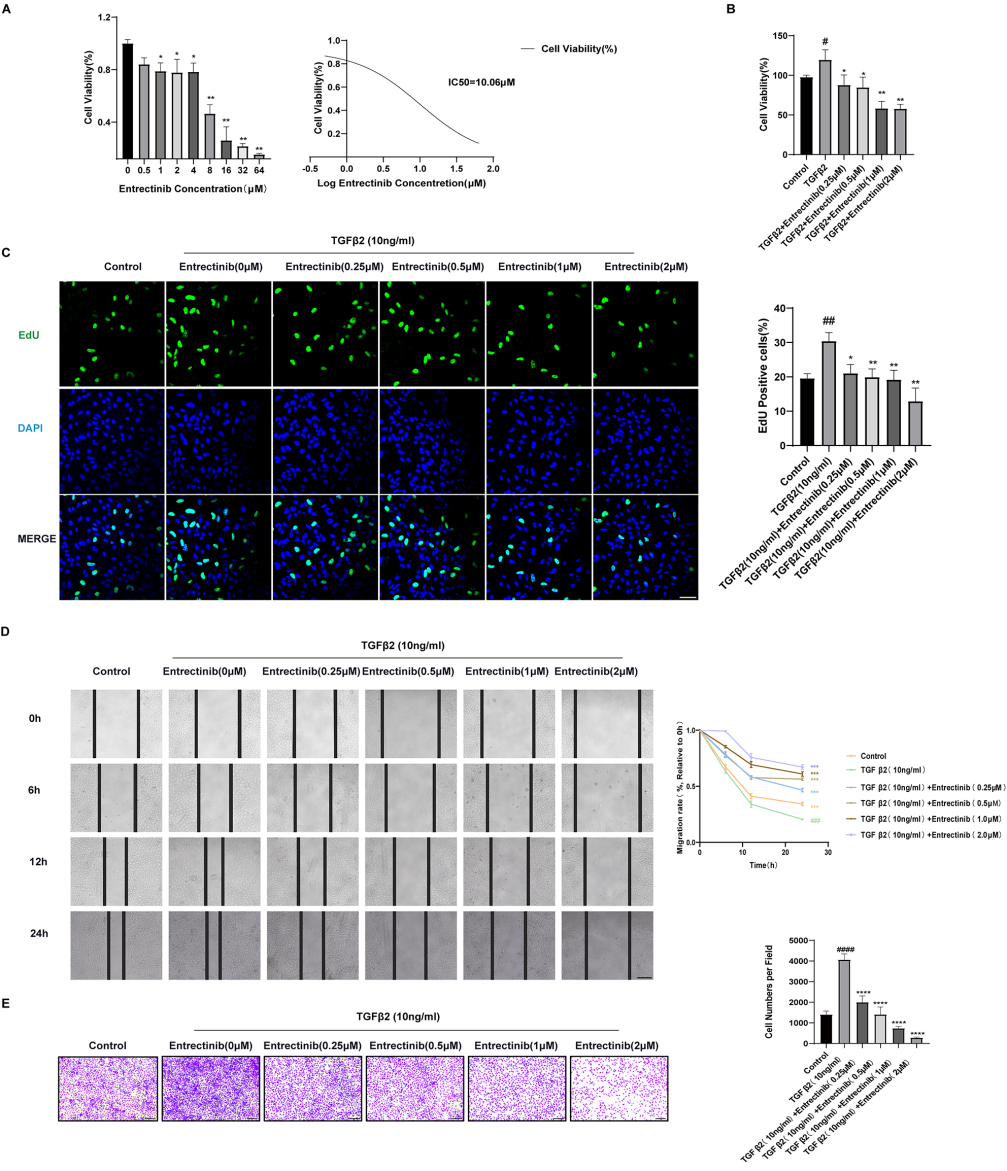
Entrectinib retards the migration and proliferation of human lens epithelial cells. (**A**) Cells were exposed to different doses of Entrectinib(0–64µM). IC50 = 10.06µM. (**B**) The effects of Entrectinib (0.25, 0.5, 1, 2µM) on proliferation induced by TGFβ2(10ng/ml) were assessed by CCK8 assay. (**C**) The EdU assay illustrated the effects of Entrectinib(0.25, 0.5, 1, 2µM) on proliferation induced by TGFβ2(10ng/ml). The proportion of EdU-positive cells was quantified and statistically analyzed (Scale bar = 200 μm). (**D**) Cell migration was recorded at 0, 6, 12, and 24 h after administrated TGFβ2(10ng/ml) and different doses of Entrectinib (0.25, 0.5, 1, 2µM). Black straight lines showed the wound edges. The migration rates were quantified and statistically analyzed (Scale bar = 200 μm). (**E**) After being seeded for 24 h, cells that moved vertically and stuck to the polycarbonate membrane’s exterior were stained with crystal violet and recorded. The number of cells that migrated through the membrane was quantified and statistically analyzed (Scale bar = 200 μm). Data was expressed as means ± SD, *n* = 3. ^**#**^*P* < 0.05, ^**##**^*P* < 0.01, ^**###**^*P* < 0.001 and ^**####**^*P* < 0.0001versus control group. ^*^P < 0.05, ^**^P < 0.01, ^***^P < 0.001 and ^****^P < 0.0001 versus TGFβ2 group

Firstly, the cytotoxicity of Entrectinib was evaluated by CCK8, and the results indicated that 10.06µM is the inhibitory concentration of 50% (IC50) of the drug on ihLECs (Fig. 3A). Stimulated by TGFβ2, ihLECs underwent EMT which aggravates the migration and proliferation. To evaluate the inhibitory effect of Entrectinib on proliferation, the CCK-8 assay and EdU assay were carried out. The increased cell viability caused by TGFβ2 can be suppressed by Entrectinib (Fig. 3B). The EdU assay also revealed that Entrectinib can inhibit TGFβ2-induced proliferation (Fig. 3C). Meanwhile, the results of the Wound Healing assay showed that, in a dose-dependent way, Entrectinib can block the horizontal migration driven on by TGFβ2 (Fig. 3D). Similarly, as demonstrated by the Transwell assay, Entrectinib can likewise, in a dose-dependent way, inhibit TGFβ2-induced vertical migration (Fig. 3E). Overall, 0.25µM of Entrectinib can inhibit the migration and proliferation of ihLECs caused by TGFβ2, and with the increase of drug concentration, the curative effect is more significant.

### Entrectinib hinders the TGFβ2-induced EMT of ihLECs

**Fig. 4:**
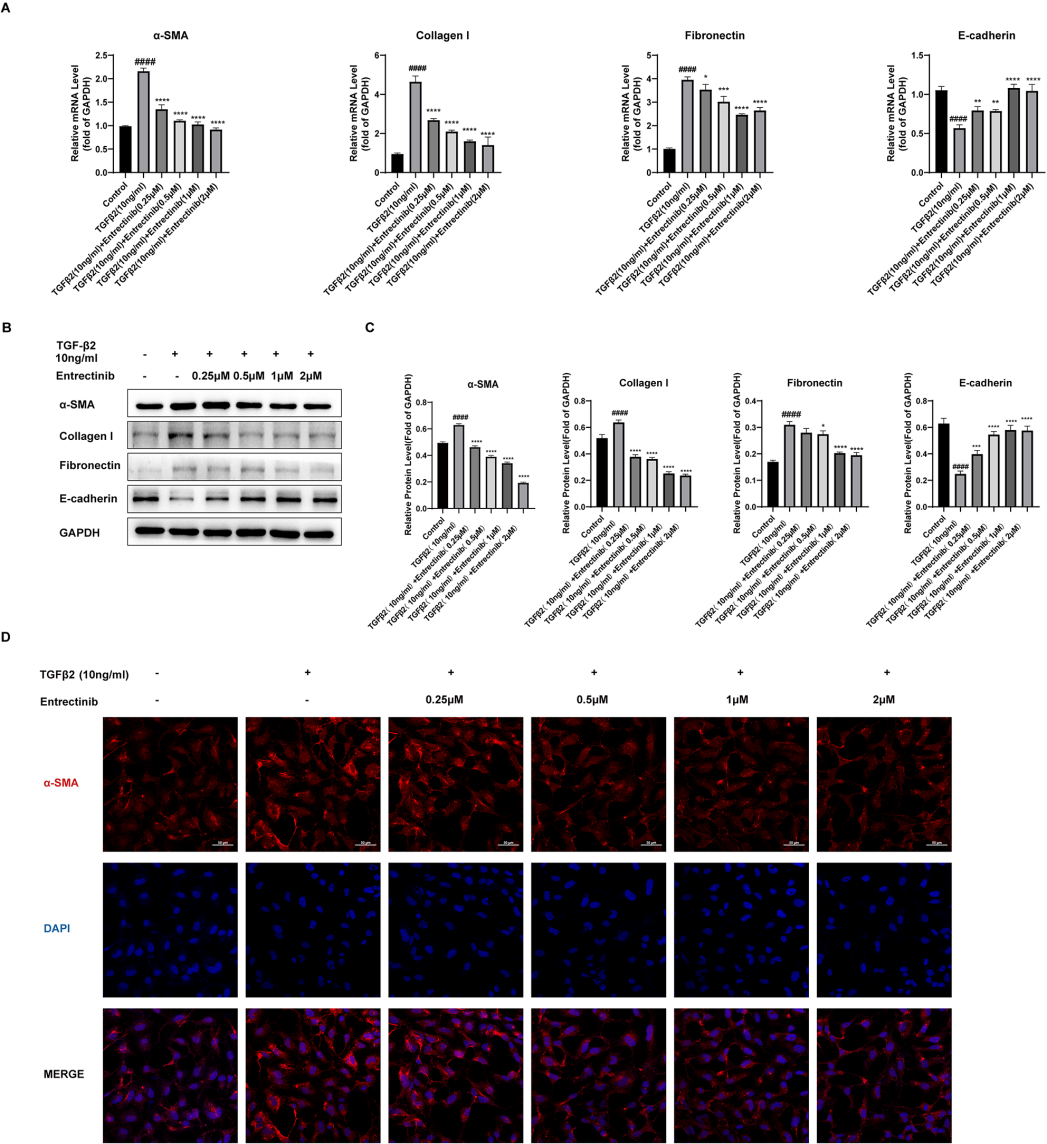
Entrectinib suppressed the EMT of the human lens epithelial cells. (**A**) Relative mRNA expression of α-SMA, collagen I, fibronectin, and E-cadherin were analyzed by quantitative real-time PCR. GAPDH served as a reference gene. (**B**-**C**) Western blot was applied to analyze the relative protein expression of α-SMA, collagen I, fibronectin, and E-cadherin in cells. GAPDH served as a reference protein. (**D**) Immunofluorescence staining of α-SMA (red) and nuclei (blue) (Scale bar = 50 μm). Data was expressed as means ± SD, *n* = 3. ^*####*^*P* < 0.0001 versus control group. ^*^P < 0.05, ^**^P < 0.01, ^***^P < 0.001, ^****^P < 0.0001 versus TGFβ2 group

To verify the anti-EMT effects of Entrectinib in vitro, we tested the expression of epithelial and fibrotic markers in ihLECs at mRNA and protein levels. Entrectinib (0.25, 0.5, 1, 2µM) can block the EMT developed in cells stimulated by TGFβ2. At the mRNA level, α-SMA, collagen I, and fibronectin expression were decreased, while E-cadherin expression was increased after Entrectinib treatment (Fig. 4A). Protein-level data showed similar trends. Fibrotic proteins, including α-SMA, collagen I, and fibronectin, were down-regulated, while E-cadherin was up-regulated (Fig. 4B-C). Immunofluorescence, in addition to qRT-PCR and WB, provided direct evidence that Entrectinib can lower α-SMA expression in the cytoplasm (Fig. 4D). Essentially, whether at mRNA or protein levels, Entrectinib exhibited a dose-dependent inhibition of EMT in vitro.

### Entrectinib exerts efficacy by inhibiting the TGFβ2/Smad and non-smad signaling pathway

**Fig. 5:**
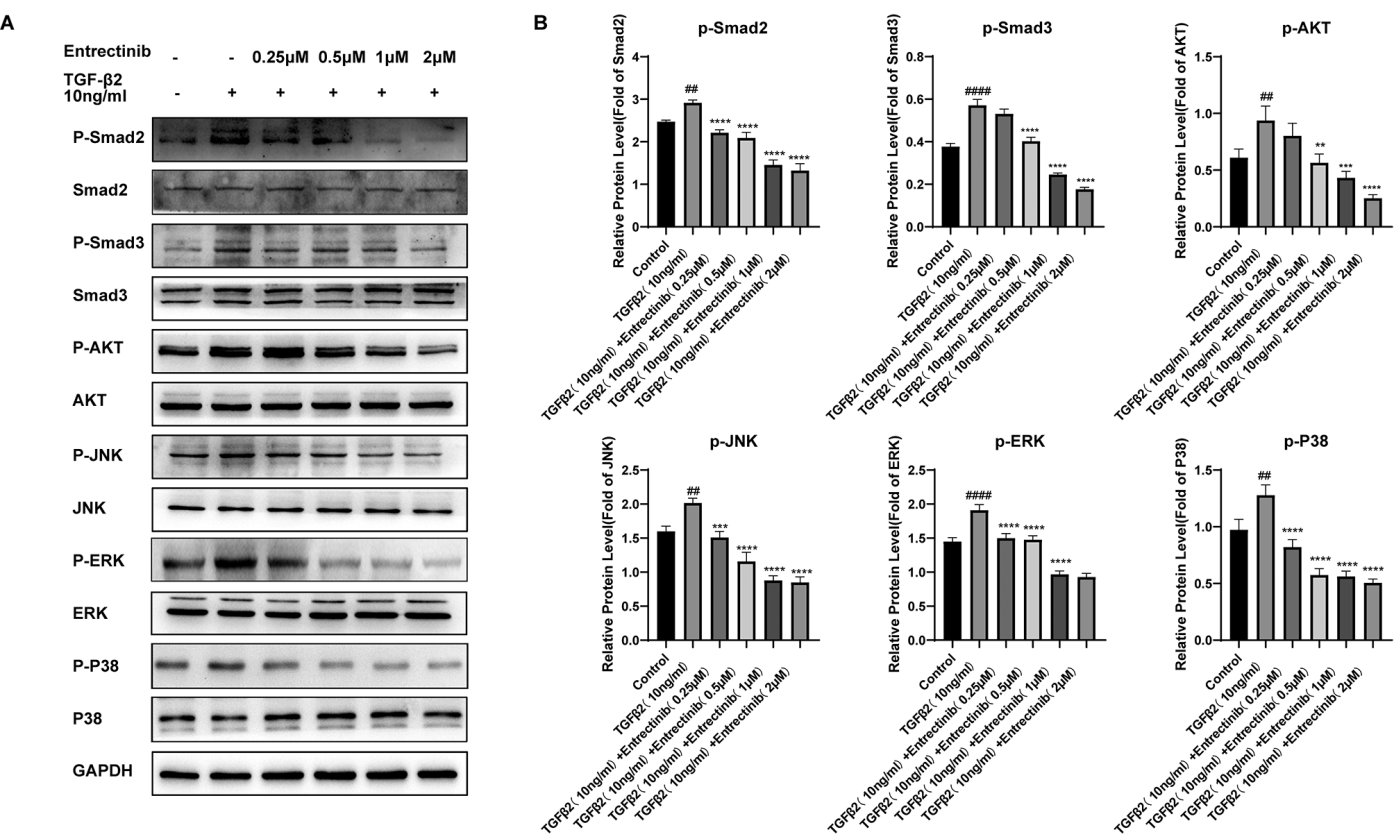
Entrectinib inhibits the activation of Smad and non-Smad signaling pathway induced by TGFβ2. (**A**-**B**) The phosphorylation levels of Smad2, Smad3, AKT, JNK, ERK, and P38 in cells treated by TGFβ2(10ng/ml) and different doses of Entrectinib (0.25, 0.5, 1, 2µM) were analyzed by western blot. GAPDH served as a reference protein. Data was expressed as means ± SD, *n* = 3. ^*#*^*P* < 0.05, ^*##*^*P* < 0.01 and ^*####*^*P* < 0.0001 versus control group. ^**^P < 0.01, ^***^P < 0.001 and ^****^P < 0.0001 versus TGFβ2 group

The essential cytokine to promote the EMT of ihLECs is TGFβ2. Thus, we further explored how Entrectinib relieves the stimulation of TGFβ2. We first studied the impact of Entrectinib on TGFβ2/Smad2/3 axis, the classic signaling pathway of TGFβ2. We discovered that Entrectinib (0.25, 0.5, 1, 2µM) can block the pathway by down-regulating the phosphorylation of Smad2 and the blocking effect was dose-dependent (Fig. 5A-B). Additionally, TGFβ2/non-Smad pathway also plays a regulatory role in organ fibrosis. In consequence, we tested if Entrectinib can hinder TGFβ2/non-Smad pathway. We discovered that Entrectinib (0.25, 0.5, 1, 2µM) can, in a dose-dependent way, reduce the phosphorylation of JNK, ERK, and P38 induced by TGFβ2. Likewise, the TGFβ2-induced phosphorylation of Smad3 and AKT can be decreased by Entrectinib, however, the efficacy is not statistically significant at 0.25µM concentration (Fig. 5A-B). In summary, in ihLECs, Entrectinib suppressed the EMT by obstructing TGFβ2/Smad2/3 and TGFβ2/non-Smad signaling pathways.

### PYK2 is a candidate target of entrectinib

**Fig. 6:**
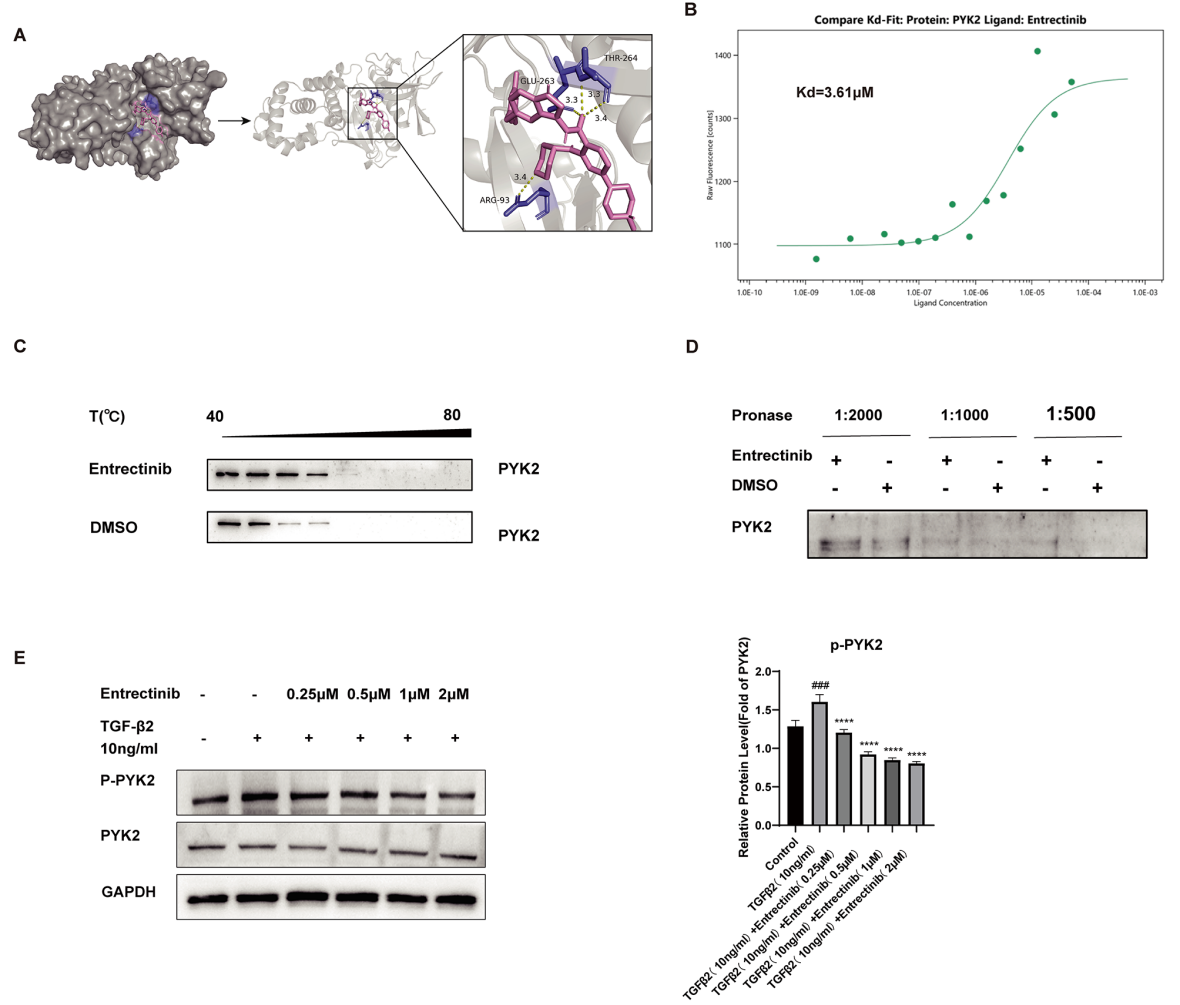
PYK2 is a potential target of Entrectinib. (**A**) The binding model between Entrectinib (pink) and the crucial residues of PYK2 (purple). (**B**) The binding affinity between PYK2 and Entrectinib was analyzed by Microscale thermophoresis (MST). (**C**) Cellular Thermal Shift Assay and Western blot were performed to evaluate the stability of PYK2 after incubation with or without Entrectinib at different temperatures. (**D**) Drug Affinity Responsive Target Stability and Western blot were performed to evaluate the resistance of PYK2 to enzymatic hydrolysis. (**E**) The phosphorylation levels of PYK2 in cells treated by TGFβ2(10ng/ml) and different doses of Entrectinib (0.25, 0.5, 1, 2µM) were analyzed by western blot. GAPDH served as a reference protein. Data was expressed as means ± SD, *n* = 3. ^*###*^*P* < 0.001 versus control group. ^****^P < 0.0001 versus TGFβ2 group

The potential targets of Entrectinib were detected on the Super-PRED( https://prediction.charite.de/index.php ) website. There is a 96% possibility that protein tyrosine kinase 2 beta (PYK2) will bind to Entrectinib. PYK2 is a non-receptor tyrosine kinase that belongs to the FAK (focal adhesion kinase) subfamily. Phosphorylated PYK2 can bind to the proteins that contain the SH2 domain, regulating the migration and proliferation of cells(Gil-Henn et al. 2023). The bonds between PYK2 and Entrectinib were evaluated by AutoDockVina software and the docking score between Entrectinib with PYK2 is -9.4 kcal/mol (Affinity). The docking mode is shown in Fig. 6A, the hydrogen bond interactions are formed between residues of GLU-263, THR-264, and ARG-93 with Entrectinib, indicating that PYK2 is a direct target of Entrectinib. Through MST, the binding affinity between PYK2 and Entrectinib was evaluated. The equilibrium dissociation constant (Kd) value was calculated as 3.61µM (Fig. 6B). Moreover, DARTS assay was further applied to validate the combination between PYK2 and Entrectinib. Compared to DMSO, Entrectinib treatment can improve PYK2 resistance to hydrolysis of pronase (Fig. 6C-D). In contrast to soluble protein solution incubated with DMSO, the stability of PYK2 in solution incubated with Entrectinib was well maintained as the temperature increased. We next explored the effects of Entrectinib on PYK2 and found that Entrectinib prevented TGFβ2-induced PYK2 phosphorylation (Fig. 6E).

### Entrectinib inhibits PYK2 activity to alleviate fibrotic cataract

**Fig. 7:**
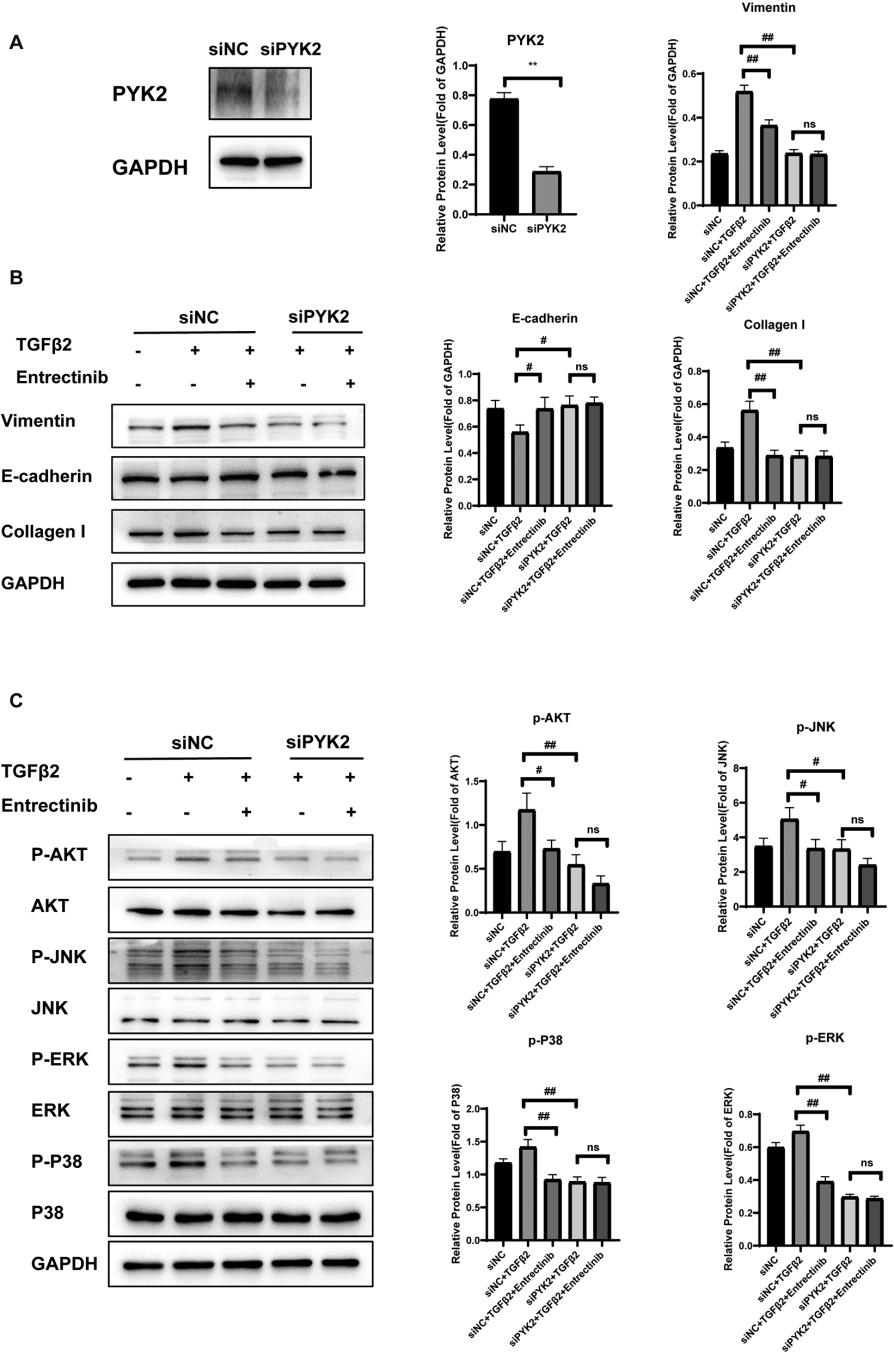
The knockdown of PYK2 inhibits the TGFβ2-induced EMT through a non-Smad signaling pathway. (**A**) The knockdown efficiency of PYK2 was evaluated by western blotting. (**B**) The knockdown of PYK2 inhibited the EMT induced by TGFβ2. The efficacy of Entrectinib is influenced by PYK2 knockdown. (**C**) PYK2 knockdown inhibited the activation of AKT, JNK, ERK, and P38. GAPDH served as a reference protein. Data was expressed as means ± SD, *n* = 3. ^*#*^*P* < 0.05 and ^*##*^*P* < 0.01 versus the control group

The phosphorylation of PYK2, a non-receptor protein tyrosine kinase, influences the activation of downstream proteins like ERK, JNK, AKT, and so on. We found that Entrectinib can suppress the phosphorylation of PYK2 by attaching to it. Furthermore, the knockdown of PYK2 can also allay the EMT by downregulating vimentin and collagen I and upregulating E-cadherin, which indicates that PYK2 is involved in fibrotic cataracts (Fig. 7A-B). Moreover, after the knockdown of PYK2, there was no significant difference between the cells treated with Entrectinib and that untreated. Subsequent studies on the phosphorylation of downstream proteins revealed that the knockdown of PYK2 hindered the activation of ERK, JNK, P38, and AKT. Additionally, the inhibitory effect cannot be significantly strengthened by the additional treatment of Entrectinib (Fig. 7C).

## Discussion

ASC and PCO seriously influence visual quality, which induces the occlusion of the optic axis and cannot be treated with drugs (Sun et al. 2021a, b; Fișuș and Findl 2020). Currently, the only way to deal with the PCO is YAG: Nd laser which may be accompanied by IOL damage, cystoid macular edema, and other complications (Maedel et al. 2021; Qin et al. 2023; Topete et al. 2021). Consequently, researchers are looking for medications that can treat fibrotic cataract. In this work, we initially evaluated the efficacy of Entrectinib in vivo and in vitro. Eventually, we certificated that Entrectinib can alleviate the EMT and fibrosis of the lens by targeting PYK2 to regulate the TGFβ2/ Smad and non-Smad signaling pathways.

Entrectinib is approved by the FDA to treat patients with NTRK-rearranged tumors and has demonstrated notable clinical success (Demetri et al. 2022; Marcus et al. 2021). Several months ago, the positive efficacy of Entrectinib on pulmonary fibrosis was reported (Miao et al. 2022). It is uncertain, nevertheless, if the medication can treat lens fibrosis. So that is necessary to explore whether Entrectinib affects fibrotic cataract. The fibrosis severity was accessed by slit lamp, H&E stain, and IF intuitively in vivo. WB and qRT-PCR were used to relatively quantify the fibrosis levels in vivo and in vitro. The mechanism of Entrectinib was also explored in vitro. Finally, we discovered that Entrectinib is a potential drug for treating lens fibrosis.

To test the efficacy of Entrectinib on fibrotic cataracts, we examined the EMT and fibrosis levels of ihLECs following medication treatment. We found that Entrectinib can mitigate the disease by inhibiting cell migration, proliferation, and the expression of ECM. In 2021, Radhika Iyer et al. corroborated that Entrectinib elevated the expression of E-cadherin, a protein extensively expressed in epithelial cells, at both mRNA and protein levels in gastric cancer cells (Sohn et al. 2021). They also proved that Entrectinib can suppress the migration of gastric cancer cells. Given that EMT is a frequent process associated with tumors and fibrosis, we explored the possibility of treating lens fibrosis with Entrectinib (Peng et al. 2022; Lee and Massagué 2022). In vitro, analogous outcomes were observed in the ihLEC stimulated by TGFβ2. Moreover, we discovered that Entrectinib may also reduce the expression of fibrotic markers, including α-SMA, collagen I, and fibronectin, which indicated that Entrectinib hindered TGFβ2-induced fibrotic progression. Besides, a prior investigation has demonstrated that Entrectinib can suppress vimentin and N-cadherin expression in mice with bleomycin-induced pulmonary fibrosis (Miao et al. 2022). ASC mouse model is a classic model for lens fibrosis study (Xiao et al. 2015; Zhang et al. 2022a, b; Jiang et al. 2023). Therefore, we applied the ASC mouse model to assess the efficacy in vivo. Based on the volume of aqueous humor, the ultimate concentration of Entrectinb in the anterior chamber ranges approximately from 1.5µM to 5.5µM, which is safe for intracameral injection. The results of the experiments in vivo showed that Entrectinib can up-regulate the expression of E-cadherin and down-regulate the expression of α-SMA, collagen I, and fibronectin at both mRNA and protein levels in ASC mouse models.

As previously stated, our studies have already certified that Entrectinib can inhibit the expression of proteins related to fibrosis, however, the mechanism of the drug is still unknown. TGFβ receptors (TβR) are dual specificity kinases. After interaction with ligands, TβRII can approach TβRI and phosphorylate the GS domain, which transfers the phosphate group to the downstream proteins (Franzén et al. 1995). The downstream proteins are abundant. Previous research demonstrated that Entretinib can slow the progression of EMT in tumor and organ fibrosis by suppressing MAPK and AKT activation (Miao et al. 2022; Sohn et al. 2021). It is recognized that MAPK and PI3K/AKT signaling pathways are also essential for the development of fibrotic cataracts (Kayastha et al. 2015; Kubo et al. 2017; Lovicu et al. 2016; Khotskaya et al. 2017). Therefore, we tested the effectiveness of Entrectinib on the relevant signaling pathways. Consistent with previous studies, the phosphorylation of ERK, P38, JNK, and AKT can be inhibited by Entrectinib. Interestingly, the phosphorylation of Smad2/3 was also suppressed by Entrectinib, which has not been manifested in previous studies. Xiaoyun Chen and colleagues have demonstrated the inactivation of ERK1/2 signaling blocked TGFβ/Smad signaling pathway (Chen et al. 2014). Some scholars have also elucidated that the ERK1/2 signaling can regulate Smad2/3 activity (Lovicu et al. 2016; Zhu et al. 2017). Thus, we inferred that the suppression of ERK1/2 activation by Entrectinib affected Smad2/3 phosphorylation. Although Entrectinib was approved by the FDA as an inhibitor of TRK1/2/3, ALK, and ROS which are mainly expressed in the nervous system, other targets can be inhibited by Entrectinib as concentration increases (Roskoski 2020). The inhibitory concentration of Entrectinib against TRK1/2/3, ROS1, and ALK is less than 2 nM, significantly less than the concentration that we applied for cells and mice (González-Sales et al. 2021). In this study, we injected Entrectinib into the anterior chamber as opposed to delivering it orally. It should be noted that this method does not require first elimination so that can achieve high drug concentrations locally. Consequently, additional targets may be acted on. Also, the drugs in the anterior chamber were eliminated by aqueous circulation, iris, and ciliary body, so the drug concentration in the blood circulation is much lower than that of oral administration (Gautam et al. 2023).

Furthermore, we explored the target of the drug in the management of fibrotic cataracts. According to the prediction of Entrectinib targets, we speculated that PYK2 is a potential one. PYK2 is a non-receptor protein-tyrosine kinase that plays a pivotal role in cell polarization and migration (Duong et al. 1998; Müller et al. 2022). In addition, PYK2 is a crucial kinase to activate the PI3K/AKT and MAPK signaling pathways (Sun et al. 2008; Lev et al. 1995; Tokiwa et al. 1996; Zhu et al. 2018) and PYK2 phosphorylation can be upregulated in liver fibrosis and renal fibrosis (Kim et al. 2020; Sonomura et al. 2012). It has also been documented that TGFβ1-stimulated hepatic stellate cells exhibited an upregulation of PYK2 phosphorylation (Kim et al. 2020). TGFβ1, as well as TGFβ2, belongs to the TGFβ family (Sun et al. 2021a, b). As the protein structure and sequence are homologous, they share the same receptors and trigger the same signaling pathways (Wang et al. 2023). Likewise, we evaluated the influence of TGFβ2 on PYK2 in ihLECs. We noted that TGFβ2 can raise the PYK2 phosphorylation and the variation can be inhibited by Entrectinb. To confirm our speculation that PYK2 is a potential target of Entrectinib, Molecular Docking was further done to simulate the interaction between Entrectinib and PYK2 (Trott and Olson 2010). The affinity score between Entrectinib and PYK2 is -9.4 kcal/mol. Meanwhile, we validated the bind between Entrectinib and PYK2 by DARTS, CETSA, and MST assays. All results indicated that Entrectinib did interact with PYK2. To further certify the involvement of PYK2 in fibrotic cataracts, we knocked down the PYK2 by siRNA and found that the knockdown of PYK2 can allay the TGFβ2-induced EMT by suppressing the phosphorylation of ERK, JNK, P38, and AKT. In addition, after the knockdown of PYK2, the additional treatment of Entrectinib can further inhibit the fibrosis, however, the differences were not significant. TGFβ2 is not the only signaling pathway that PYK2 is involved. PYK2 and FAK are the only two members of the FAK family. They show a high degree of homology (Kim et al. 2020). Research by Liu Jie et al. has proved that both TGFβ2 and integrin can activate FAK in regulating the cell migration and EMT of LECs (Liu et al. 2018). Previous scholars have verified that PYK2 can also be activated by integrin signaling when EMT takes place (Nakamura et al. 2001). It may be deduced that PYK2 is triggered by various upstream signals, including TGFβ2 and integrin, then phosphorylates downstream pathways, affecting cell migration and EMT. Therefore, Entrectinib may affect the integrin signaling pathway by binding with PYK2 to relieve fibrotic cataract, which should be further explored. To sum up, PYK2 is an important target of Entrectinib in the treatment of fibrotic cataract.

The study does, of course, have certain limitations. The injury-induced ASC mouse model is currently a widely used animal model in the study of fibrotic cataract, however, more research is still needed to fully understand the pharmacokinetics of this paradigm. In addition, extended-release formulations of Entrectinib may have better therapeutic effects, which requires further interdisciplinary collaboration for in-depth research.

## Conclusion

In conclusion, we demonstrated that Entrectinib alleviates lens fibrosis and depresses myofibroblast activation by targeting PYK2 to inhibit the TGFβ2/Smad and non-Smad signaling pathways. Entrectinib is a prospective drug candidate for fibrotic lens treatment.

## Data Availability

Data is available on request from the authors. Data will be made available on request

## References

[CR16] Al-Salama ZT, Keam SJ, Entrectinib. First Global Approval Drugs. 2019;79(13):1477–83. 31372957 10.1007/s40265-019-01177-y

[CR3] Berthoud VM, Minogue PJ, Osmolak P, Snabb JI, Beyer EC. Roles and regulation of lens epithelial cell connexins. FEBS Lett. 2014;588(8):1297–303. 24434541 10.1016/j.febslet.2013.12.024 PMC3992928

[CR45] Chen X, Ye S, Xiao W, Wang W, Luo L, Liu Y. ERK1/2 pathway mediates epithelial-mesenchymal transition by cross-interacting with TGFβ/Smad and Jagged/Notch signaling pathways in lens epithelial cells. Int J Mol Med. 2014;33(6):1664–70. 24714800 10.3892/ijmm.2014.1723

[CR19] Demetri GD, De Braud F, Drilon A, Siena S, Patel MR, Cho BC, et al. Updated Integrated Analysis of the efficacy and safety of Entrectinib in patients with NTRK Fusion-positive solid tumors. Clin Cancer Res. 2022;28(7):1302–12. 35144967 10.1158/1078-0432.CCR-21-3597 PMC9365368

[CR17] Doebele RC, Drilon A, Paz-Ares L, Siena S, Shaw AT, Farago AF, et al. Entrectinib in patients with advanced or metastatic NTRK fusion-positive solid tumours: integrated analysis of three phase 1–2 trials. Lancet Oncol. 2020;21(2):271–82. 31838007 10.1016/S1470-2045(19)30691-6 PMC7461630

[CR18] Drilon A, Siena S, Dziadziuszko R, Barlesi F, Krebs MG, Shaw AT, et al. Entrectinib in ROS1 fusion-positive non-small-cell lung cancer: integrated analysis of three phase 1–2 trials. Lancet Oncol. 2020;21(2):261–70. 31838015 10.1016/S1470-2045(19)30690-4 PMC7811790

[CR50] Duong LT, Lakkakorpi PT, Nakamura I, Machwate M, Nagy RM, Rodan GA. PYK2 in osteoclasts is an adhesion kinase, localized in the sealing zone, activated by ligation of alpha(v)beta3 integrin, and phosphorylated by src kinase. J Clin Invest. 1998;102(5):881–92. 9727056 10.1172/JCI3212 PMC508953

[CR20] Dziadziuszko R, Krebs MG, De Braud F, Siena S, Drilon A, Doebele RC, et al. Updated Integrated Analysis of the efficacy and safety of Entrectinib in locally Advanced or Metastatic ROS1 Fusion-positive non-small-cell Lung Cancer. J Clin Oncol. 2021;39(11):1253–63. 33646820 10.1200/JCO.20.03025 PMC8078299

[CR27] El Deeb S, Al-Harrasi A, Khan A, Al-Broumi M, Al-Thani G, Alomairi M et al. Microscale thermophoresis as a powerful growing analytical technique for the investigation of biomolecular interaction and the determination of binding parameters. Methods Appl Fluoresc. 2022;10(4). 10.1088/2050-6120/ac82a6 35856854

[CR22] Fan Y, Zhang B, Du X, Wang B, Yan Q, Guo L et al. Regulating tumorigenicity and Cancer metastasis through TRKA Signaling. Curr Cancer Drug Targets. 2023. 10.2174/1568009623666230904150957 37670705

[CR30] Fișuș AD, Findl O. Capsular fibrosis: a review of prevention methods and management. Eye (Lond). 2020;34(2):256–62. 31804626 10.1038/s41433-019-0723-5 PMC7002601

[CR21] Frampton JE, Entrectinib. A review in NTRK + solid tumours and ROS1 + NSCLC. Drugs. 2021;81(6):697–708. 33871816 10.1007/s40265-021-01503-3 PMC8149347

[CR40] Franzén P, Heldin CH, Miyazono K. The GS domain of the transforming growth factor-beta type I receptor is important in signal transduction. Biochem Biophys Res Commun. 1995;207(2):682–9. 7864860 10.1006/bbrc.1995.1241

[CR49] Gautam M, Gupta R, Singh P, Verma V, Verma S, Mittal P, et al. Intracameral Drug Delivery: a review of agents, indications, and outcomes. J Ocul Pharmacol Ther. 2023;39(2):102–16. 36757304 10.1089/jop.2022.0144

[CR28] Gil-Henn H, Girault J-A, Lev S. PYK2, a hub of signaling networks in breast cancer progression. Trends Cell Biol. 2023. 10.1016/j.tcb.2023.07.006 37586982

[CR48] González-Sales M, Djebli N, Meneses-Lorente G, Buchheit V, Bonnefois G, Tremblay P-O et al. Population pharmacokinetic analysis of entrectinib in pediatric and adult patients with advanced/metastatic solid tumors: support of new drug application submission. Cancer Chemother Pharmacol. 2021;88(6). 10.1007/s00280-021-04353-8 34536094

[CR7] Gotoh N, Perdue NR, Matsushima H, Sage EH, Yan Q, Clark JI. An in vitro model of posterior capsular opacity: SPARC and TGF-beta2 minimize epithelial-to-mesenchymal transition in lens epithelium. Invest Ophthalmol Vis Sci. 2007;48(10):4679–87. 17898292 10.1167/iovs.07-0091

[CR5] Imaizumi T, Kurosaka D, Tanaka U, Sakai D, Fukuda K, Sanbe A. Topical administration of a ROCK inhibitor prevents anterior subcapsular cataract induced by UV-B irradiation. Exp Eye Res. 2019;181:145–9. 30690025 10.1016/j.exer.2019.01.016

[CR39] Jiang F, Qin Y, Yang Y, Li Z, Cui B, Ju R, et al. BMP-4 and BMP-7 inhibit EMT in a model of Anterior Subcapsular Cataract in Part by regulating the Notch Signaling Pathway. Invest Ophthalmol Vis Sci. 2023;64(4):12. 37043340 10.1167/iovs.64.4.12 PMC10103728

[CR24] Jin W. Roles of TrkC signaling in the regulation of Tumorigenicity and Metastasis of Cancer. Cancers (Basel). 2020;12(1). 10.3390/cancers12010147 PMC7016819 31936239

[CR12] Kanasi E, Ayilavarapu S, Jones J. The aging population: demographics and the biology of aging. Periodontol 2000. 2016;72(1):13–8. 27501488 10.1111/prd.12126

[CR41] Kayastha F, Johar K, Gajjar D, Arora A, Madhu H, Ganatra D, et al. Andrographolide suppresses epithelial mesenchymal transition by inhibition of MAPK signalling pathway in lens epithelial cells. J Biosci. 2015;40(2):313–24. 25963259 10.1007/s12038-015-9513-9

[CR44] Khotskaya YB, Holla VR, Farago AF, Mills Shaw KR, Meric-Bernstam F, Hong DS. Targeting TRK family proteins in cancer. Pharmacol Ther. 2017;173:58–66. 28174090 10.1016/j.pharmthera.2017.02.006

[CR56] Kim J, Kang W, Kang SH, Park SH, Kim JY, Yang S, et al. Proline-rich tyrosine kinase 2 mediates transforming growth factor-beta-induced hepatic stellate cell activation and liver fibrosis. Sci Rep. 2020;10(1):21018. 33273492 10.1038/s41598-020-78056-0 PMC7713048

[CR42] Kubo E, Shibata S, Shibata T, Kiyokawa E, Sasaki H, Singh DP. FGF2 antagonizes aberrant TGFβ regulation of tropomyosin: role for posterior capsule opacity. J Cell Mol Med. 2017;21(5):916–28. 27976512 10.1111/jcmm.13030 PMC5387175

[CR36] Lee JH, Massagué J. TGF-β in developmental and fibrogenic EMTs. Semin Cancer Biol. 2022;86(Pt 2):136–45. 36183999 10.1016/j.semcancer.2022.09.004 PMC10155902

[CR53] Lev S, Moreno H, Martinez R, Canoll P, Peles E, Musacchio JM, et al. Protein tyrosine kinase PYK2 involved in ca(2+)-induced regulation of ion channel and MAP kinase functions. Nature. 1995;376(6543):737–45. 7544443 10.1038/376737a0

[CR8] Li Q, Liu S, Yang G, Li M, Qiao P, Xue Q. Naringenin inhibits autophagy and epithelial-mesenchymal transition of human lens epithelial cells by regulating the Smad2/3 pathway. Drug Dev Res. 2022;83(2):389–96. 34402084 10.1002/ddr.21868

[CR61] Liu J, Xu D, Li J, Gao N, Liao C, Jing R, et al. The role of focal adhesion kinase in transforming growth factor-β2 induced migration of human lens epithelial cells. Int J Mol Med. 2018;42(6):3591–601. 30280182 10.3892/ijmm.2018.3912

[CR6] Liu ZZG, Taiyab A, West-Mays JA. MMP9 differentially regulates proteins involved in actin polymerization and Cell Migration during TGF-β-Induced EMT in the Lens. Int J Mol Sci. 2021;22(21). 10.3390/ijms222111988 PMC8584335 34769418

[CR2] Liu Z, Huang S, Zheng Y, Zhou T, Hu L, Xiong L, et al. The lens epithelium as a major determinant in the development, maintenance, and regeneration of the crystalline lens. Prog Retin Eye Res. 2023;92:101112. 36055924 10.1016/j.preteyeres.2022.101112

[CR43] Lovicu FJ, Shin EH, McAvoy JW. Fibrosis in the lens. Sprouty regulation of TGFβ-signaling prevents lens EMT leading to cataract. Exp Eye Res. 2016;142. 10.1016/j.exer.2015.02.004 PMC4654713 26003864

[CR14] Maedel S, Evans JR, Harrer-Seely A, Findl O. Intraocular lens optic edge design for the prevention of posterior capsule opacification after cataract surgery. Cochrane Database Syst Rev. 2021;8(8):CD012516. 34398965 10.1002/14651858.CD012516.pub2 PMC8406949

[CR33] Marcus L, Donoghue M, Aungst S, Myers CE, Helms WS, Shen G, et al. FDA approval Summary: Entrectinib for the treatment of NTRK gene Fusion Solid tumors. Clin Cancer Res. 2021;27(4):928–32. 32967940 10.1158/1078-0432.CCR-20-2771

[CR26] Miao Y, Li X, Yang Y, Zhang J, Chen L, Zhang Q, et al. Entrectinib ameliorates bleomycin-induced pulmonary fibrosis in mice by inhibiting TGF-β1 signaling pathway. Int Immunopharmacol. 2022;113Pt B:109427. 10.1016/j.intimp.2022.109427 36375321

[CR23] Moriwaki K, Wada M, Kuwabara H, Ayani Y, Terada T, Higashino M, et al. BDNF/TRKB axis provokes EMT progression to induce cell aggressiveness via crosstalk with cancer-associated fibroblasts in human parotid gland cancer. Sci Rep. 2022;12(1):17553. 36266462 10.1038/s41598-022-22377-9 PMC9584965

[CR51] Müller A-K, Köhler UA, Trzebanski S, Vinik Y, Raj HM, Girault J-A, et al. Mouse modeling dissecting macrophage-breast Cancer Communication uncovered roles of PYK2 in macrophage recruitment and breast tumorigenesis. Adv Sci (Weinh). 2022;9(9):e2105696. 35092356 10.1002/advs.202105696 PMC8948556

[CR62] Nakamura K, Yano H, Schaefer E, Sabe H. Different modes and qualities of tyrosine phosphorylation of Fak and Pyk2 during epithelial-mesenchymal transdifferentiation and cell migration: analysis of specific phosphorylation events using site-directed antibodies. Oncogene. 2001;20(21):2626–35. 11420674 10.1038/sj.onc.1204359

[CR35] Peng D, Fu M, Wang M, Wei Y, Wei X. Targeting TGF-β signal transduction for fibrosis and cancer therapy. Mol Cancer. 2022;21(1):104. 35461253 10.1186/s12943-022-01569-x PMC9033932

[CR31] Qin C, Wen S, Fei F, Han Y, Wang H, Chen H, et al. NIR-triggered thermosensitive polymer brush coating modified intraocular lens for smart prevention of posterior capsular opacification. J Nanobiotechnol. 2023;21(1):323. 10.1186/s12951-023-02055-2 PMC10483730 37679734

[CR47] Roskoski R. Properties of FDA-approved small molecule protein kinase inhibitors: a 2020 update. Pharmacol Res. 2020;152:104609. 31862477 10.1016/j.phrs.2019.104609

[CR34] Sohn S-H, Sul HJ, Kim BJ, Kim HS, Zang DY. Entrectinib induces apoptosis and inhibits the epithelial-mesenchymal transition in gastric Cancer with NTRK overexpression. Int J Mol Sci. 2021;23(1). 10.3390/ijms23010395 PMC8745632 35008821

[CR57] Sonomura K, Okigaki M, Kimura T, Matsuoka E, Shiotsu Y, Adachi T, et al. The kinase Pyk2 is involved in renal fibrosis by means of mechanical stretch-induced growth factor expression in renal tubules. Kidney Int. 2012;81(5):449–57. 22157654 10.1038/ki.2011.403

[CR25] Su J, Morgani SM, David CJ, Wang Q, Er EE, Huang Y-H, et al. TGF-β orchestrates fibrogenic and developmental EMTs via the RAS effector RREB1. Nature. 2020;577(7791):566–71. 31915377 10.1038/s41586-019-1897-5 PMC7450666

[CR10] Sugiyama Y, Nakazawa Y, Sakagami T, Kawata S, Nagai N, Yamamoto N, et al. Capsaicin attenuates TGFβ2-induced epithelial-mesenchymal-transition in lens epithelial cells in vivo and in vitro. Exp Eye Res. 2021;213:108840. 34798144 10.1016/j.exer.2021.108840

[CR52] Sun CK, Man K, Ng KT, Ho JW, Lim ZX, Cheng Q, et al. Proline-rich tyrosine kinase 2 (Pyk2) promotes proliferation and invasiveness of hepatocellular carcinoma cells through c-Src/ERK activation. Carcinogenesis. 2008;29(11):2096–105. 18765415 10.1093/carcin/bgn203

[CR29] Sun Y, Xiong L, Wang X, Wang L, Chen B, Huang J, et al. Autophagy inhibition attenuates TGF-β2-induced epithelial-mesenchymal transition in lens epithelial cells. Life Sci. 2021a;265:118741. 33181173 10.1016/j.lfs.2020.118741

[CR58] Sun T, Huang Z, Liang W-C, Yin J, Lin WY, Wu J, et al. TGFβ2 and TGFβ3 isoforms drive fibrotic disease pathogenesis. Sci Transl Med. 2021b;13:605. 10.1126/scitranslmed.abe0407 34349032

[CR4] Taiyab A, West-Mays J. Lens Fibrosis: understanding the dynamics of Cell Adhesion Signaling in Lens epithelial-mesenchymal transition. Front Cell Dev Biol. 2022;10:886053. 35656546 10.3389/fcell.2022.886053 PMC9152183

[CR54] Tokiwa G, Dikic I, Lev S, Schlessinger J. Activation of Pyk2 by stress signals and coupling with JNK signaling pathway. Science. 1996;273(5276):792–4. 8670418 10.1126/science.273.5276.792

[CR32] Topete A, Saramago B, Serro AP. Intraocular lenses as drug delivery devices. Int J Pharm. 2021;602:120613. 33865952 10.1016/j.ijpharm.2021.120613

[CR60] Trott O, Olson AJ. AutoDock Vina: improving the speed and accuracy of docking with a new scoring function, efficient optimization, and multithreading. J Comput Chem. 2010;31(2):455–61. 19499576 10.1002/jcc.21334 PMC3041641

[CR13] Wang W, Yan W, Fotis K, Prasad NM, Lansingh VC, Taylor HR, et al. Cataract Surgical Rate and Socioeconomics: A Global Study. Invest Ophthalmol Vis Sci. 2016;57(14):5872–81. 27802517 10.1167/iovs.16-19894

[CR9] Wang L, Tian Y, Shang Z, Zhang B, Hua X, Yuan X. Metformin attenuates the epithelial-mesenchymal transition of lens epithelial cells through the AMPK/TGF-β/Smad2/3 signalling pathway. Exp Eye Res. 2021;212:108763. 34517004 10.1016/j.exer.2021.108763

[CR59] Wang M-Y, Liu W-J, Wu L-Y, Wang G, Zhang C-L, Liu J. The Research Progress in transforming growth Factor-β2. Cells. 2023;12(23). 10.3390/cells12232739 PMC10706148 38067167

[CR11] Wormstone IM, Wormstone YM, Smith AJO, Eldred JA. Posterior capsule opacification: what’s in the bag? Prog Retin Eye Res. 2021;82:100905. 32977000 10.1016/j.preteyeres.2020.100905

[CR37] Xiao W, Chen X, Li W, Ye S, Wang W, Luo L, et al. Quantitative analysis of injury-induced anterior subcapsular cataract in the mouse: a model of lens epithelial cells proliferation and epithelial-mesenchymal transition. Sci Rep. 2015;5:8362. 25666271 10.1038/srep08362 PMC4322358

[CR1] Xiong L, Sun Y, Huang J, Ma P, Wang X, Wang J et al. Long non-coding RNA H19 prevents Lens Fibrosis through maintaining Lens epithelial cell phenotypes. Cells. 2022;11(16). 10.3390/cells11162559 PMC9406623 36010635

[CR15] Zhang Y, Zhang C, Chen S, Hu J, Shen L, Yu Y. Research Progress concerning a novel intraocular Lens for the Prevention of posterior capsular opacification. Pharmaceutics. 2022a;14(7). 10.3390/pharmaceutics14071343 PMC9318653 35890240

[CR38] Zhang L, Wang L, Hu X-B, Hou M, Xiao Y, Xiang J-W, et al. MYPT1/PP1-Mediated EZH2 dephosphorylation at S21 promotes epithelial-mesenchymal transition in fibrosis through Control of Multiple Families of Genes. Adv Sci (Weinh). 2022b;9(14):e2105539. 35293697 10.1002/advs.202105539 PMC9108659

[CR46] Zhu Y, Gu J, Zhu T, Jin C, Hu X, Wang X. Crosstalk between Smad2/3 and specific isoforms of ERK in TGF-β1-induced TIMP-3 expression in rat chondrocytes. J Cell Mol Med. 2017;21(9):1781–90. 28230313 10.1111/jcmm.13099 PMC5571561

[CR55] Zhu X, Bao Y, Guo Y, Yang W. Proline-Rich Protein Tyrosine Kinase 2 in inflammation and Cancer. Cancers (Basel). 2018;10(5). 10.3390/cancers10050139 PMC5977112 29738483

